# Poultry Farms as a Potential Source of Environmental Pollution by Pharmaceuticals

**DOI:** 10.3390/molecules25051031

**Published:** 2020-02-25

**Authors:** Katarzyna Wychodnik, Grażyna Gałęzowska, Justyna Rogowska, Marta Potrykus, Alina Plenis, Lidia Wolska

**Affiliations:** 1 Department of Environmental Toxicology, Faculty of Health Sciences with Institute of Maritime and Tropical Medicine, Medical University of Gdansk, Debowa 23A St., 80-204 Gdańsk, Poland; k.wychodnik@gumed.edu.pl (K.W.); grazyna.galezowska@gumed.edu.pl (G.G.); justyna.rogowska@gumed.edu.pl (J.R.); marta.potrykus@gumed.edu.pl (M.P.); 2 Department of Pharmaceutical Chemistry, Faculty of Pharmacy, Medical University of Gdansk, Hallera 107 St., 80-416 Gdansk, Poland; aplenis@gumed.edu.pl

**Keywords:** pharmaceuticals, soil, poultry farms, ultra-high performance liquid chromatography, antibiotics, antibiotic resistance

## Abstract

Industrial poultry breeding is associated with the need to increase productivity while maintaining low meat prices. Little is known about its impact on the environment of soil pollution by pharmaceuticals. Breeders routinely use veterinary pharmaceuticals for therapeutic and preventive purposes. The aim of this work was to determine the influence of mass breeding of hens on the soil contamination with 26 pharmaceuticals and caffeine. During two seasons—winter and summer 2019—15 soil samples were collected. Liquid extraction was used to isolate analytes from samples. Extracts were analyzed using ultra-high performance liquid chromatography coupled with tandem mass spectrometry detection (UPLC-MS/MS). The results showed the seasonal changes in pharmaceutical presence in analyzed soil samples. Ten pharmaceuticals (metoclopramide, sulphanilamide, salicic acid, metoprolol, sulphamethazine, nimesulide, carbamazepine, trimethoprim, propranolol, and paracetamol) and caffeine were determined in soil samples collected in March, and five pharmaceuticals (metoclopramide, sulphanilamide, sulphamethazine, carbamazepine, sulfanilamid) in soil samples collected in July. The highest concentrations were observed for sulphanilamide, in a range from 746.57 ± 15.61 ng/g d.w to 3518.22 ± 146.05 ng/g d.w. The level of bacterial resistance to antibiotics did not differ between samples coming from intensive breeding farm surroundings and the reference area, based on antibiotic resistance of 85 random bacterial isolates.

## 1. Introduction

Pharmaceuticals are a group of compounds designed to elicit specific biological effects at relatively low concentrations. These compounds can be used to treat both humans and animals. According to Regulation (EU) 2019/6 of the European Parliament and of the Council of 11 December, 2018, on veterinary medicinal products, and repealing Directive 2001/82/EC (OJ L 4, 7.1.2019, p. 43–167), “veterinary medicinal product” means any substance or combination of substances that has properties for treating or preventing disease in animals; or its purpose is to be used in, or administered to animals, with a view to restoring, correcting, or modifying physiological functions by exerting a pharmacological, immunological, or metabolic action. The definition also includes pharmaceutical products used on animals to make a medical diagnosis and in euthanasia of animals [1]. More than 2000 veterinary pharmaceutical products are manufactured from 400 active chemical ingredients to treat various species of animals [2]. Veterinary pharmaceuticals prevent and treat disease and increase the efficiency of food production [3]. These pharmaceuticals belong to several pharmacological categories: antiparasitics (ectoparasiticides, endectocides, and endoparasiticides, including antiprotozoals and anthelmintics), antimicrobials, hormones, antifungals, anti-inflammatory (steroidal and non-steroidal drugs), anaesthetics, tranquilizers, sedatives, bronchodilators, antacids, diuretics, and emetics [4]. The largest group are antimicrobial agents. Antimicrobials are compounds that can kill or inhibit the growth of microorganisms (bacteria, archaea, protozoa, microalgae, and fungi) [5]. Some of these drugs also show growth-promoting effects and are commonly misused for this reason [6].

Pharmaceuticals, massively used in veterinary medicine, can end up in the environment via several routes. Consumed drugs, including antimicrobial agents, are continuously discharged into the natural ecosystems via excretion (urine and feces) after a short time of residence in animal organisms. That main sources of veterinary pharmaceuticals in the environment are intensive livestock activities and farming practices [7]. The agglomeration of large numbers of birds in a small area causes the need for pharmaceuticals, including anti-microbial agents, to prevent and treat microbial infections, as well as to increase feed efficiency [8]. Moreover, to prevent and control contagious poultry diseases vaccines are used. This results in lower bird mortality and, thus, increases the profitability of breeding.

The production of poultry results in hatchery wastes, manure (bird excrement), litter (bedding materials such as sawdust, wood shavings, straw, and peanut or rice hulls), and on-farm mortalities [9]. Intensive livestock farming practices that are used to breed thousands of poultry, often in small areas, face problems with safe and proper disposal of tons of animal excreta produced every day [2]. The practice of using manure for soil fertilization purposes is the major contributor of veterinary pharmaceutical contamination in the environment [10]. These may cause a raise in antibiotic resistance of microbial strains isolated from chickens. Braykov et al. [11] showed that the number of resistant strains isolated from poultry bred in intensive breeding farms is much larger than the number of resistant strains isolated from henhouses. Poultry farms are the biggest emitters of dust, microorganisms, and organic compounds (including pharmaceuticals) in manure, litter, dust, and air [12]. Odor emissions, consisting of a large number of compounds, including ammonia, volatile organic compounds (VOCs), and hydrogen sulfide, adversely affect the life of people living in the vicinity of poultry farms [13]. Dust is one of the components present in poultry production; it originates from poultry residues, molds, and feathers, and is biologically active as it contains microorganisms, some of which may be pathogens [14]. Consequently, fertilization with antibiotics containing animal manure, dust, and sewage sludge seems to be the likely pathway for the release of antibiotics into soil [2]. Moreover, spiking animal manure with antibiotics and applying it on soil showed changes in microbial soil composition and rising antibiotic resistance of soil microbial community [15]. Additionally, antibiotics can migrate from soil to groundwater, or into plants; thus, can cause negative consequences for human health [16].

Commercial livestock production has increased rapidly in the past few years, which has promoted the construction of large production units. The world leader in intensive poultry breeding is the United States of America. In Europe, Poland is the main shareholder in terms of poultry production. In addition, it should be noted that gross domestic production of poultry meat in Poland almost doubled between 2012 and 2017 (from 1,712,000 to 3,110,000 tons carcass weight) [17]. Poland is the only European Union (EU) country in which such significant growth has been observed. The number of farms involved in industrial animal husbandry and the scale of the impact on the environment is different in individual regions of Poland. The results of the audit, carried out by the Supreme Audit Office in 2011–2013, showed the lack of proper supervision by administrative authorities over the functioning of animal farms, ineffective cooperation between the Veterinary Inspection and the Inspection of Environmental Protection in the field of control, and a lack of legislation aimed at solving the problem of odors caused by farms [18]. Veterinary Inspection bodies, and to some extent, the State Sanitary Inspection, are obliged to constantly monitor the presence of prohibited substances and antibiotic resistance in food products of animal origin, as well as to supervise the marketing and use of pharmaceuticals in the process of animal breeding [19]. The audit carried out by the Supreme Audit Office in 2015–2016 regarding the use of antibiotics in animal production showed that the scale of use of antibiotics in animal production is not exactly known in Poland. This is because the Ministry of Agriculture and Rural Development only has data provided by pharmaceutical wholesalers on the number of veterinary antibiotics sold on the domestic market. At the same time, it was found that the lack of the above-mentioned data is due to the lack of a nationwide system for collecting, monitoring, and comparing data on the use of antibiotics at farm level. The problem was also related to deficiencies in the documentation regarding animal treatment [19]. In 2015–2017, the Supreme Audit Office carried out supervision over the marketing and use of products containing anabolic, hormonal, narcotic, and psychotropic substances in the treatment of animals in Poland. As a result, it was found that both the Veterinary Inspection and Pharmaceutical Inspection activities are insufficient to limit the risk of using these substances contrary to their intended use. The audit found use of drugs not in accordance with their intended use, discrepancies as to the drugs used, or their dose [20].

In effect, an unidentified number of pharmaceuticals can migrate into the environment, including soils. Therefore, it is necessary to study the pharmaceutical content in soils around poultry farms.

The aim of the study was to determine whether mass breeding of hens influences the contamination level of the surrounding soil with pharmaceuticals. The choice of analyzed compounds resulted from the information obtained from the breeder and the review of literature on the routine use of pharmaceuticals in the process of intensive breeding. The first stage of research was to develop a procedure for isolating and determining the selected pharmaceuticals. In the next step, the method was validated. In the last stage, the content of pharmaceuticals in soil samples taken around the poultry farmhouse was determined. Additionally, the prevalence of antibiotic resistant bacterial strains in the soil samples taken around the poultry house was verified.

## 2. Materials and Methods

### 2.1. Study Area

#### 2.1.1. Weather Conditions

The farm is located in one of the communes in the northeastern part of Poland. Geographic location translates into specific climate conditions. The warmest and the sunniest month is July (average temperature 17 °C). It is coldest in January (average temperature −1.7 °C). The highest rainfall is recorded in July (85 mm) and the lowest in March (33 mm). Winds from the west and southwest sectors dominate in this area. The average wind speed is 3 m/s, with the highest winds occurring in the winter and the lowest in the summer [21].

In March, on the day of sampling, it was sunny and rainless and the humidity was 55%. The air temperature was around 6–7 °C. The wind was negligible (2–4 m/s), southwest, and spread along the shed wall. In July, on the day of sampling, the weather was mostly sunny, with temporary cloud cover, rainless, and humidity was 47%–58%. The air temperature was between 17 and 21 °C. West wind gusts up to 8.3 m/s.

#### 2.1.2. Poultry House

**Figure 1:**
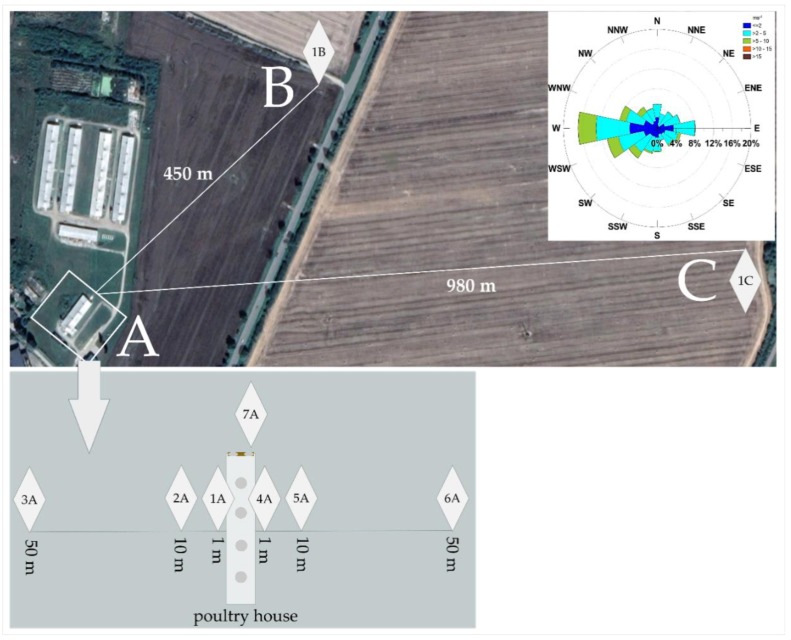
Soil sampling point distribution. 1–7A, 1B, and 1C soil samples point, collected in area A (around poultry house), area B (450 m away), and area C (980 m away).

The whole farm consists of five poultry houses and two outbuildings. Four of the poultry houses are large with an area of 1700 m^2^, and one is smaller, at a distance around 80 m from them. The impact of the smaller poultry house, on the soil, was the object of this study (Figure 1). The farm is engaged in broiler breeding. The chicks are bought from an external company, which provides the necessary vaccines and injections of a lincomycin and spectinomycin mixture. The breeding cycle lasts 42 days (6 weeks) with the initial temperature of 31 °C, falling down to 18 °C during the breeding, and constant air circulation (supply, exhaust, heating). Food given to the chickens is supplied in the form of a mixture of soybean meal, wheat, triticale, corn, soybean oil, and premix as a supplement (ready mix of minerals and vitamins, contains 2.5 kg of salt per ton of additives). However, during breeding, the necessary amount of antibiotics (such as amoxicillin, doxycycline, and enrofloxacin) are given ad hoc in water troughs (the line is not cleaned after supply). Notwithstanding, the antibiotic withdrawal period is respected, under threat of punitive measures by the meat purchasing company.

According to the breeder, proceedings involving distant discharge of polluted air and its high frequency are to maintain hygiene in the poultry house itself, and eliminate the controversial odor around the farm. The poultry house is cleaned once, before the start of every cycle (one cycle = 42 breeding days) by an external company. As part of cleaning, the litter is replaced, and the surface is swept and wet-cleaned each time with the use of cleaning agents, fired and whitewashed with lime, with the addition of ammonium sulfate for disinfection. Before settling the shed, disinfectant is sprayed in mist form. The composition of cleaning and disinfecting agents is unknown, and at the same time considered irrelevant in these tests. After replacement, the litter is moved outside the building at point 7A_M and then taken outside the farm.

### 2.2. Sample Handling

Soil samples were collected on the farm area (samples 1–7A). Six sampling locations, three on each of the two sides of the poultry house, were selected by measuring 1, 10, and 50 m from the longer wall. One sample was collected near the litter storage place. Aside from the samples in the area of direct impact of the poultry house, reference samples were taken (Figure 1). The reference sample 1B was taken at a distance of 450 m in a straight line to the North East. The references sample 1C was taken at a distance of about 1000 m in a straight line to the East. Samples were taken twice (in March, samples suffix M, and in July samples suffix J) to check for potential seasonal variation, in both cases, about 5 weeks after the poultry house was settled.

Using a shovel, about 1 L of soil was collected from each point at a depth of about 20 cm. In each case, the collected soil material was transported to the laboratory and stored in glass containers at 4 °C. Before sample preparation, each sample passed through a 1.5 mm-mesh sieve (to remove roots and stones) and was freeze-dried using the Scanvac CoolSafe 110-4 PRO, LyoAlfa (Telstar, Denmark).

For microorganisms’ determination in soil samples, the samples were collected into sterile plastic containers. The samples were stored at 10 °C until processing, which was done within 3 days after samples collection.

### 2.3. Analytical Procedure

#### 2.3.1. Solvents and Standards

Methanol, acetonitrile (LiChrosolv^®^Hypergrade) were purchased from Merck (Darmstadt, Germany). Isopropanol was purchased from Sigma-Aldrich (Saint Louis, MO, USA). Formic acid 98%–100% pure (LiChrosolv^®^Hypergrade) to acidify the mobile phases and extraction solutions was purchased from Merck (Darmstadt, Germany). Ultra-pure water was produced using Hydrolab system (Hydrolab, Straszyn, Poland).

All pharmaceutical standards were of high purity (for HPLC analysis). Most of the native standards were purchased from Sigma-Aldrich (Saint Louis, MO, USA): ampicillin (AMP), caffeine (CAF), carbamazepine (CBZ), ciprofloxacin (CIP), diclofenac (DIC), enrofloxacin (ENR), ibuprofen (IB), metoclopramide (MTC), metoprolol (MET), nimesulide (NIM), paracetamol (PAR), propranolol (PROP), salicylic acid (SA), acetylsalicylic acid (ASA), sulfacetamide (SFC), spectinomycin (SPEC), streptomycin (STREP), sulfacarbamide (SCA), sulfadiazine (SDA), sulfaguanidine (SGA), sulfamerazine (SMA), sulfamethazine (SMZ), sulfamethoxazole (SMX), sulfanilamide (SNA), sulfathiazole (STA), tetracycline (TET) and trimethoprim (TMP).

#### 2.3.2. Sample Pretreatment

Soil samples were prepared by weighing 8 g of dry soil (from each sample, separately) in 20 mL vials. Samples were extracted using two-stage extraction [22]. In the first step, a mixture of acetonitrile and water (1:1 *v*/*v*) with the addition of 0.1% formic acid was used. The second stage of extraction consisted of using a mixture of acetonitrile, 2-propanol, and water (3:3:4 *v*/*v*/*v*), also with the addition of 0.1% formic acid. For both the 1st and 2nd stage of extraction, 2 mL of extraction solvent was added for every 1 g of soil—16 mL of solvent for every step. The soil and solvent were mixed using a vortex mixer for 30 s, and then subjected to ultrasound using an IS-5,5 ultrasonic cleaner (Intersonic, Olsztyn, Poland) for 15 min. The decanted supernatant, collected after each of the steps, was filtered through a syringe filter (hydrophilic PTFE 0.2 μm membrane, Merck Millipore, (Tullagreen, Carrigtohill, Co. Cork, Ireland) into 40 mL glass vials. The filtered extracts from both steps were combined (in total, 32 mL) and evaporated to dryness under a stream of nitrogen. The residue was dissolved in 2 mL mixture of 25% methanol in water with the addition of 0.1% formic acid.

In order to eliminate the possible effect of the matrix, the same procedure of extraction was carried out with the addition of pharmaceutical standards (corresponding to a current-like concentration of compounds in soil). The mixture of the standards was added to 8 g of soil from each sample point. The rest of the procedure was carried out in accordance with the above-described method.

#### 2.3.3. UPLC-MS/MS Analysis

The prepared samples were analyzed by ultra-performance liquid chromatography. The Nexera X2UPLC-MS/MS (Shimadzu Corp., Kyoto, Japan) contained two LC-30AD pumps, SiL-30AC autosampler, CTO-20AC column oven, CBM-20A communication bus module, and mass spectrometer LC-MS8050 with electro spray ionization with positive and negative ion mode (Table 1). Chromatographic separation was performed using an analytical column Kinetex 2.6 µm, Phenyl-Hexyl 100 Å 4.6 mm, 150 mm (Phenomenex, Torrance, CA, USA). The gradient program consisted of the following: 7-min sequence of linear gradient flows of solvent B (acetonitrile:methanol 1:1 *v*/*v*) balanced with solvent A (water with 0.1% formic acid) at a flow rate of 0.6 mL/min: 50–80% B over 1 min, 80–100% B over 2 min, isocratic 100% B for 3 min, and finally, 100–50% B over 1 min. The injection volume was 1 µL and column temperature was 40 °C.

The main parameters used to identify analytes were their retention times and multiple reaction monitoring (MRM) ratio, which were obtained at 0.25 µg/mL working standard solutions for most standards, and 2.5 µg/mL for AMP, IB, SPEC, STREP, SFC, SNA and TET. Chromatographic data processing was carried out using LabSolution^®^ software (Shimadzu Corp., Kyoto, Japan).

#### 2.3.4. Quality Assurance/Quality Control (QA/QC)

Ensuring the quality of the measurements results was carried out by:

- calibration of the LC-MS/MS system,
- determination of the selected validation parameters based on calibration curves.

The calibration step involves the preparation of calibration curves and calculation of response factor (RF) for internal standards. Eleven working standard solutions have been prepared. Limit of determination (LOD) has been calculated from the parameters of a calibration curve constructed on the basis of the three lowest concentrations of working standards solutions. The limit of determination has been calculated according to the formula:

(1) $$LOD=\frac{3.3\times s}{a}$$

where: a—slope of the calibration curve; s—the standard deviation.

For the calculation of the validation parameters, residual standard deviation (sxy) and standard deviation of the intercept (sa) were taken. Based on LOD(sxy) and LOD(sa) the mean values (LOD) were calculated (see Equation (1)). The coefficients of variation (CV) and uncertainty (U) were calculated based on standard deviations and the average area of the chromatographic peaks obtained during separation of standard mixtures.

To check the matrix effect, two samples were prepared using real soil samples with addition of standards, and using the procedure described in Section 2.3.2. Sample Pretreatment and additionally using solide phase extraction (SPE) cleaning. Based on peak areas, the recovery (%) for standard solutions was calculated.

### 2.4. Antibiotic Resistance in Microorganisms Isolated from Soil

The soil samples (1–6A; 1B and 1C, Figure 1) were plated on different growth media for evaluation of microbial growth (Luria Bertani Agar—LA, MacConkey Agar, Chapman Agar, Medium with cetrimide, Merck, Darmstadt, Germany). Briefly, 10 g of the sample was mixed with 90 mL of 0.85% NaCl and shaken for 0.5 h, and then serially diluted to 10^−4^. In addition, 100 µL of each dilution was plated on LA, while solely the dilution 10^−1^ was spread on MacConkey Agar, Chapman Agar and Medium with cetrimide. The plates were incubated at 30 °C for 24–72 h to obtain sufficient growth of the colonies. From each sample, several differently-looking colonies were picked and grown to pure cultures on LA, and then stored frozen with 20% glycerol at −40 °C.

The isolated bacteria were then subjected to antibiotic resistance disc diffusion test with different antibiotics (TMP 5 µg, SMZ 20 µg, TET 10 µg, SPEC 25 µg, and CIP 10 µg). TET, SPEC, and CIP discs were purchased from Oxoid, UK, while trimethoprim and sulfametazine discs were prepared in the laboratory. Briefly, the cotton discs were cut from MN85/220 paper filters (Macherey-Nagel, Germany) and autoclaved. The appropriate amount of the antibiotic stock solution was poured onto the paper disk with automatic pipette and let dry. For the antibiotic resistance disc test, the bacteria were cultured in LB (Luria Bertani boullion, Btl, Łódź, Poland) with 150 rpm shaking at 30 °C, overnight. Then, the OD_600_ measurements of the bacterial cultures were taken with Spectroquant Prove 600 (Merck, Darmstadt, Germany) spectrophotometer and diluted in LB to achieve OD_600_ equal to 0.1. The dilution accurateness was verified with repeated spectrophotometer measurements. In addition, 100 µL of each bacterial suspension was spread on a plate with Muiller-Hinton medium (Graso, Owidz, Poland). Discs with antibiotics were then put on the surface of the plate and the plates were incubated for 24 h at 30 °C. Afterwards, the diameter of the growth inhibition zone around each antibiotic disc was measured.

## 3. Results

### 3.1. Quality Assurance/Quality Control (QA/QC)

Based on analysis of working standard solutions in optimal conditions, calibration curves were prepared. Regression coefficients and selected validation parameters are presented in Table 2. The obtained results are satisfactory, showing that the proposed method is suitable for analysis of the selected pharmaceuticals. The recovery for standards were calculated based on peak areas. Recoveries ranged from 65% to 121%, for all pharmaceuticals (mean values for individual compounds: ASA-65%, AMP-81%, CAF-111%, CMZ-98%, CIP-102%, DIC-67%, ENR-111%, IB-92%, MTC-99%, MET-121%, NIM-102%, PAR-81%, PROP-115%, SA- 78%, SFC-75%, SPEC-71%, STREP-74%, SFC-82%, SDA-89% SGA-94% SMA-98%, SMZ-100%, SMX-106%, SNA-98%, STA-82%, TET-67%, TMP-79%). The recoveries of analytes were dependent on the matrix type. This suggests that the use of standard addition or analysis with labeled standards are necessary in order to improve the correction results.

### 3.2. Analysis of Real Samples

**Figure 2:**
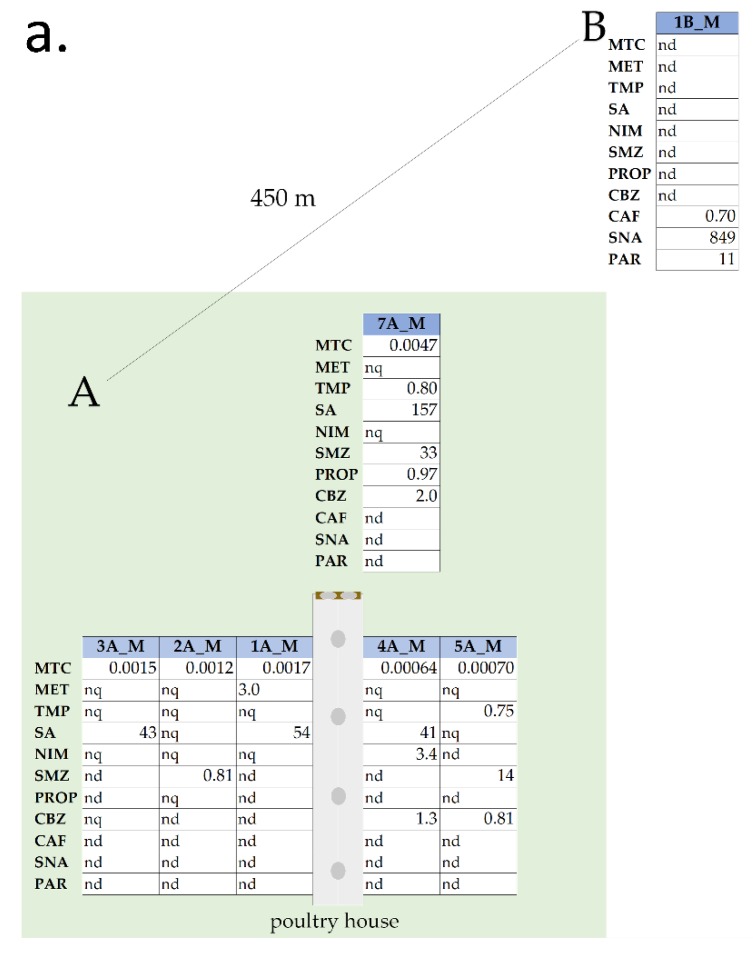

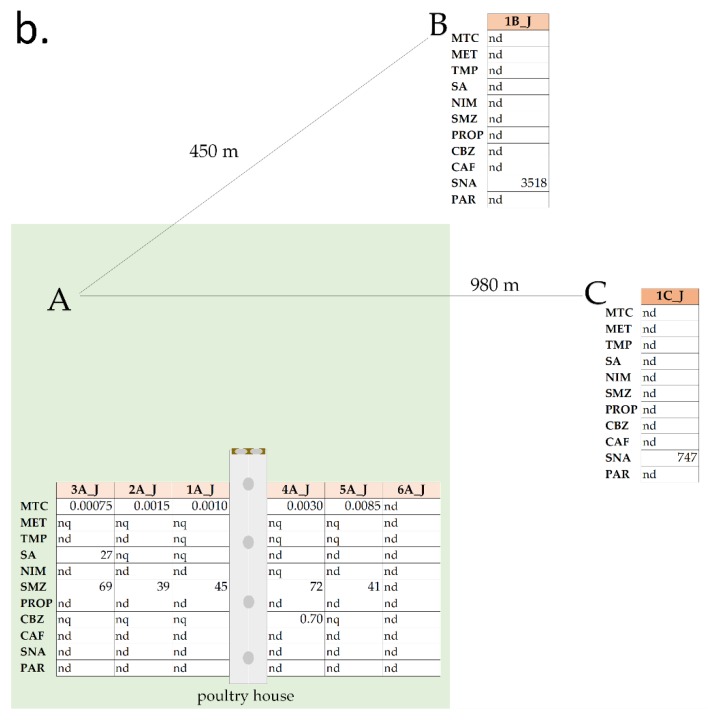
Concentration of detected pharmaceuticals and caffeine in soil samples (ng/g d.w); (**a**) 1–7A_M, 1B_M, soil samples, collected in area A (around poultry house) and area B (450 m away) in March (M); (**b**) 1–7A_J, 1B_J, 1C_J soil samples, collected in area A (around poultry house) area B (450 m away) and area C (980 m away) in July (J). MTC—matoclopramide, MET—metoprolol, TMP—trimpetoprim, SA—salicic acid, NIM—nimesulide, SMZ—supfamethazine, PROP—propranolol, CBZ—carbamazepine, CAF—caffeine, SNA—sulfanilamide, PAR—paracetamol.

Using the developed and validated method described above, a chromatographic analysis of extracts of real soil samples was performed. Each of the 15 samples was analyzed for the presence of 27 substances. Of these, 10 pharmaceuticals (MTC, SNA, SA, MET, SMZ, NIM, CBZ, TMP, PROP, PAR) and CAF were determined in soil samples collected in March (Figure 2a) and five pharmaceuticals (MTC, SA, SMZ, CBZ, SNA) in soil samples collected in July (Figure 2b). The largest number of various pharmaceuticals (MTC, TMP, SA, SMZ, PROP, CBZ) was determined at 7A_M (the sample collected in March, location where the dirty litter was thrown away). None of the 27 pharmaceuticals sought was determined in sample 6A_J. In reference samples (1B_M, 1B_J and 1C_J) two of the pharmaceuticals (PAR, SNA) and CAF were determined, which were not found in the farm area. Moreover, none of the pharmaceuticals determined on the farm area were determined in reference samples. Therefore, farm area samples (area A) and reference location samples (area B and C) will be described separately, below. 

#### 3.2.1. Area A

MTC was determined in each of the tested samples around the farm, regardless of the sampling season (except point 6A_J). However, the concentrations in the samples were significantly lower—ranging from 4.69 × 10^−3^ ± 0.16 ng/g d.w in 7A_M to 7.08×10^−4^ ± 0.263 ng/g d.w in 5A_M. Similarly, SMZ was present in eight samples, and was determined in relatively high concentration levels. The range was from 68.70 ± 1.74 ng/g d.w. in 3A_J, to 0.81 ± 0.19 ng/g d.w in 2A_M. SA was detected in a range from 157.30 ± 4.65 ng/g d.w in 7A_M, to 27.19 ± 6.01 ng/g d.w in 3A_J. CBZ was found in four samples, three of which were collected in March. The highest concentration of CBZ was determined in sample 7A_M and amounted to 1.96 ± 0.17 ng/g d.w. In turn, the lowest concentration of CBZ was determined in the July 4A_J sample, with concentration equal to 0.70 ± 0.26 ng/g d.w. TMP was detected only in two samples from March on a similar level (7A_M 0.80 ± 0.03 ng/g d.w, 5A_M 0.75 ± 0.02 ng/g d.w.). The other four determined pharmaceuticals were found in individual soil samples. NIM was determined in the 4A_M sample in a concentration level of 3.43 ± 0.03 ng/g d.w., MET in the 1A_M sample at a concentration level of 2.99 ± 0.04 ng/g d.w., and PROP in sample 7A_M at a concentration level equal to 0.97 ± 0.005 ng/g d.w.

#### 3.2.2. Areas B and C

As was mentioned, three other pharmaceuticals were found in the reference samples: CAF, PAR, and SNA. CAF (0.70 ± 0.13 ng/g d.w.) and PAR (11.31 ± 0.29 ng/g d.w.) were determined only in sample 1B_M. In turn, SNA was determined in all three reference samples with the highest concentration (3518.22 ± 146.05 ng/g d.w.) in sample 1B_J. In sample 1B_M, which is the winter counterpart of 1B_J, the concentration of SNA was 848.83 ± 1.19 ng/g d.w. In sample 1C_J, SNA was determined in the concentration level of 746.57 ± 3.90 ng/g d.w.

### 3.3. Analysis of Microbial Resistance in Soil Samples

The bacterial communities able to grow from soil samples collected around the poultry breeding building were shown to be diverse (Supplementary Materials Figure S1). From each collecting time point, a few differently-looking bacterial colonies were picked and further analyzed for antibiotic resistance. Microbial resistance of selected soil bacterial isolates was performed for five different antibiotics, two of which (TMP and SMZ) were determined in soil samples in this study. Three other antibiotics (TET, SPEC and CIP) were analyzed since they prove to be the ones that are commonly used in chicken treatment during their growth in intensive breeding farms [23,24]. The average inhibition zones for bacterial strains isolated from different sampling points situated in zones A, B and, C around the poultry breeding farm are presented in Table 3. The least susceptibility of the tested strains was observed for sulfametazine and trimethoprim, where only six and seven susceptible isolates were found in chicken farm soil, respectively. In the soil sample collected in zone B, this number was even lower, as only two strains were susceptible to TMP and none was susceptible to SMZ. In zone C, none of the strains was susceptible to TMP and SMZ; however, here the number of tested strains was only four. On the other hand, the highest susceptibility was shown for CIP (all strains from each type of soil were susceptible), and an intermediate one was shown for TET and SPEC. Taking into account the results obtained for 85 random bacterial isolates, coming from different areas around the poultry breeding building, we found no significant difference between the bacterial resistance tested for different areas (Table 3).

## 4. Discussion

There is no doubt that, especially in large-scale farms, pharmaceuticals are routinely used for both therapeutic and prophylactic purposes [8,22].

Literature reports on the presence of pharmaceuticals in soil that are potentially related to the poultry industry mainly relate to contaminants resulting from direct soil exposure to chicken droppings. Wei et al. [25] found the presence of sulfonamide and fluoroquinolone drugs in soil in areas loaded with poultry manure. Significant concentrations of sulfonamides were determined at the level of 682–1784 µg/kg soil. Jing et al. [26] presented the detection of antibiotics (tetracyclines and sulfonamides) in soils potentially exposed to chicken manure in northeast China. The concentration levels of pharmaceuticals were 0.29–1590.16 ug/kg soil, with the maximum concentration observed for chlorotetracycline.

Basic information on pharmaceuticals administrated to chickens was obtained from the breeder. According to the interview regarding the drugs used during breeding, lincomycin, SPEC, amoxycilin, doxycyclin, and ENR were administered to chickens. From the above, ENR and SPEC were selected for testing, but none of them was determined.

SMZ, together with TMP, is administered to chickens as a broad-spectrum antibiotic against most gram-negative organisms [23]. Farmers reported administrating them with water (in water line) [11]. Nevertheless, although both substances (TMP and SMZ) are given to chickens in the same quantitative ratio, their concentration level in the soil sample is significantly different. TMP is at the limit of quantification (LOQ) and has only been measured in two samples (7A_M and 5A_M), both in March. In turn, SMZ was determined in three samples collected in March, and in almost all samples collected around the poultry house in July, at higher concentration levels than in March. The difference between March and July values for SMZ can be explained by higher SMZ supply to chickens caused by greater probability of infection in July, because of the high temperature outside. Moreover, given the proportionally lower content of TMP compared to SMZ, TMP may be present in the same samples in which SMZ was determined, but below limit of quantification.

MTC is administered as an antiemetic and intestinal peristalsis drug, and must be given via subcutaneous or intramuscular injections every 12 h. MTC is given to inhibit, among others, the process of defecation—which is also intended to maintain greater hygiene in the poultry house during breeding [27]. The highest MTC concentrations were observed at 7A_M where used litter is temporarily stored. Taking all of this into consideration, it can be concluded that poultry litter is a serious source of pharmaceuticals.

The presence of SA in the environmental samples can be explained by salicylate administration to humans and/or animals, including acetylsalicylic acid (ASA) [28]. The use of salicylates has been systematically increasing for over 100 years. One route of metabolization for this group of drugs is rapid deacetylation to SA in a reaction catalized by a nonspecific enzyme. In effect, only about 68% of the dose reaches the systemic circulation as ASA, while the serum half time duration of ASA is approximately 20 min. Finally, SA and its metabolites are renally excreted [29]. So far, SA was detected in the effluent and river streams [30], and found—in low nanogram per liter concentrations—in groundwater from several areas in Ontario, Canada [31], waters from three watersheds in Nova Scotia, Canada [32], and spring water in Mexico [33]. In our research, ASA was not detected in any collected sample, but SA was determined. Moreover, SA was determined at a higher concentration, as compared to other pharmaceuticals, around the poultry house.

In addition to MTC, SMZ, TMP, and SA described earlier, PROP, CBZ were also determined. Their presence, especially in the 7A_M sample, may be associated with drug use in the process of chicken breeding.

Apart from pharmaceuticals presence in the samples, sample distribution around the farm building was considered. The obtained results show that as the distance from the poultry house increases, the concentration of pharmaceuticals increases as well. This may be due to the way of removing air from the poultry house, as fans on the roof expel air and thus spread contaminated air around the farm building.

The sample containing the largest load of pharmaceuticals was 7A_M (Figure 2). This sample was taken at the location of temporary litter storage removed from the poultry house after the end of the breeding cycle. The 7A_M sample was taken in the fifth week of a new breeding cycle, after litter removal. The presence of litter in this place before taking the sample may be the reason why 6 out of 11 substances were determined in this sample. Moreover, they were detected in the highest concentration levels compared to other samples. It can be assumed that the presence of specific pharmaceuticals in this sample may indicate their use in this breeding cycle.

Qualitative pharmaceuticals composition between the samples from farm area and reference area is diverse. The set of compounds detected in area A differs from the one detected in areas B and C. The reference sampling distance was far enough not to overlap with the poultry house impact.

The enhanced antibiotic resistance of bacterial strains was recently shown in *Escherichia coli* isolates from chicken manure originating from intensive breeding farms [24]. In regards to the presence of antibiotic resistant strains in the soil around intensive breeding farms, the information is scarce. In our study, we observed no difference between the average bacterial resistance of randomly selected soil isolates from intensive farm surroundings and agricultural soil when challenging them with five different antibiotics. However, Zhang et al. [34] observed that spiking poultry manure with antibiotics changes the average resistance in the microbiome of a soil treated with antibiotics containing manure. Indeed, the risk for soil contamination, and enhancing the number of antibiotic resistant microorganisms, may be lower in the surroundings of the intensive breeding farm than in the fields where the poultry manure was spread, as no significant difference was observed between the strains isolated in the vicinity of poultry farm buildings (area A, B, or C).

## 5. Conclusions

Soil, due to its specific features, such as sorption capacity, distribution coefficient (Kd), and hydrophobicity, is a pharmaceutical receiving matrix more durable than water [35]. Manure is one of the sources of various substances entering soil, including pharmaceuticals. Nevertheless, there is a high probability that the distribution of concentration around the poultry house (tendency-dependent changes depending on the distance) is associated with the transfer of pharmaceuticals with dust exhausted through the fans present on the building roof.

The results showed that the observed changes in pharmaceutical presence in analyzed soil samples can be defined as seasonal (Figure 2a,b). In all summer samples, less substances (five pharmaceuticals) were determined in contrast with samples collected in March (10 pharmaceuticals and CAF). This may indicate that low temperature is a parameter responsible for prolonged half-life time of determined contaminants [35]. However, the MTC concentration in both samples collected in March and July are, relatively, at a similar level. Moreover, concentration levels of SMZ and SNA in samples collected in July was approximately five times higher than in March.

Although advances in instrumentation and the opportunities that liquid chromatography methodologies bring (especially advanced ones, e.g., those coupled with tandem mass spectrometry (LC-MS/MS), facilitating the detection of even trace amounts of pharmaceuticals, in the case of environmental samples, the analyst still faces problems. The matrix effect, which is often difficult to compensate for, and with which we undoubtedly deal with in soil samples, may be the source of numerous discrepancies in qualitative assessment. The developed procedure allows separation and identification of 26 pharmaceuticals and CAF in a swift way (LC-MS/MS analysis—7 min), which proves to be an efficient way of real soil sample analysis. There is no doubt that this type of research needs to be further developed to provide a foundation for risk assessment of drugs entering the environment. It may influence human and animal health, which have been ignored in environmental monitoring programs.

## Figures and Tables

**Table 1:** The analysis parameters for the monitored ion transitions and triple quadruple mass spectrometer (MS/MS) operation (CE- collision energy, Q1 and Q3Pre Bias—quadruple pre-rod bias voltage).

	Parameters for the Monitored Ion Transition					
Name	Short	Polarity	Parent Ion > Fragment Ion	Q1 Pre Bias [V]	CE [V]	Q3 Pre Bias [V]
acetylsalicylic acid	ASA	−	179.10 > 136.95	13	11	24
ampicillin	AMP	+	349.9 > 106.05	20	25	20
caffeine	CAF	+	194.90 > 138.00	20	20	20
carbamazepine	CBZ	+	237.05 > 194.00	20	20	20
ciprofloxacin	CIP	+	322.00 > 314.10	20	21	20
diclofenac	DIC	−	294.05 > 249.90	15	12	16
enrofloxacin	ENR	+	360.10 > 316.10	20	20	20
ibuprofen	IB	−	205.00 > 161.20	14	9	16
metoclopramide	NIM	−	307.05 > 228.95	16	17	20
metoprolol	MTC	+	300.05 > 227.00	20	20	20
nimesulide	MET	+	268.15 > 116.00	20	20	20
paracetamol	PAR	+	152.00 > 110.10	17	18	21
propranolol HCl	PROP	+	260.10 > 116.10	20	19	20
salicylic acid	SA	−	137.30 > 93.15	10	17	20
spectinomycin	SPEC	+	332.80 > 98.10	16	29	14
streptomycin	STREP	+	582.00 > 263.20	28	33	11
sulfacetamide Na	SFC	+	214.95 > 92.05	11	23	21
sulfacarbamide	SCA	+	216.00 > 92.05	30	25	30
sulfadiazine	SDA	+	251.00 > 92.05	18	26	19
sulfaguanidine	SGA	+	214.95 > 92.05	15	26	21
sulfanilamide	SNA	+	172.9 > 86.15	19	16	13
sulfamerazine	SMA	+	264.95 > 92.05	13	29	14
sulfamethazine	SMZ	+	278.50 > 186.50	14	19	27
sulfamethoxazole	SMX	+	253.95 > 92.00	20	30	20
sulfathiazole	STA	+	255.90 > 156.00	20	15	20
tetracycline	TET	+	445.00 > 409.95	20	20	20
trimethoprim	TMP	+	291.05 > 230.10	20	25	20
	**MS/MS Operation Parameters**					
	Interface Temperature (°C)			300		
	Desolvation LineTemperature (°C)			250		
	Nebulizing Gas Flow (L/min)			3		
	Heating Gas Flow (L/min)			9		
	Heating Block (°C)			350		
	Drying Gas Flow (L/min)			10		

**Table 2:** Summary of validation data for quantification of selected pharmaceuticals and caffeine in environmental samples by UHPLC-MS/MS method (*n* = 6). LOD—Limit of dectection, LOQ—Limit of quantification, MDL—Method Detection Limit, MQL—Minimum Quantification Limit.

Pharmaceuticals	Short	Coefficient of Calibration Curve (y = ax + b)		Linearity		Limits				Repeatability	
		a	b	Regression Coefficient R^2^	min–max (ng/mL)	LOD (ng/mL)	LOQ (3 × LOD) (ng/mL)	MDL (ng/g)	MQL (ng/g)	CV (%)	U (*k* = 2)
acetylsalicylic acid	ASA	107058	−1301	0.9931	176.57–5000	58.86	176.57	14.72	44.15	2.5–2.8	0.95
ampicillin	AMP	663156	2850	0.9996	14.25–5000	4.75	14.25	1.19	3.56	1.1–3.9	0.78
caffeine	CAF	29004046	128181	0.999	3.9–500	0.75	2.25	0.19	0.56	0.79–3.9	0.98
carbamazepine	CMZ	17335651	9152	0.9991	8.8–500	2.93	8.80	0.73	2.20	1.0–11	0.65
ciprofloxacin	CIP	2687453	1143	0.9982	9.9–5000	3.3	9.9	0.8	2.5	1.9–11.0	1.4
diclofenac	DIC	6826340	130246	0.9984	8.86–1000	2.89	8.68	0.72	2.17	0.71–9.6	0.64
enrofloxacin	ENR	12634935	−64163	0.9974	22.4–1000	7.5	22.4	1.9	5.6	1.4–5.1	1.4
ibuprofen	IB	10369	16715	0.9912	384.37–5000	316.8	950.4	79.2	237.6	2.2–3.8	1.1
metoclopramide	MTC	14227180430	1348134	0.998	0.39 × 10^−3^	0.13 × 10^−3^	0.39 × 10^−3^	0.03 × 10^−3^	0.09 × 10^−3^	0.24–8.4	0.39
metoprolol	MET	8726542	10467	0.9991	3.3–1000	1.1	3.3	0.3	0.8	0.41–4.4	1.5
nimesulide	NIM	134064871	3435909	0.9934	12.62–500	4.21	12.62	1.05	3.16	1.6–7.5	0.60
paracetamol	PAR	1696703	1073	0.9993	14.01–5000	4.67	14.01	1.17	3.50	0.76–9.9	0.87
propranolol HCl	PROP	12583700	38410	0.9993	2.8–1000	0.92	2.8	0.2	0.7	0.26–4.6	1.2
salicylic acid	SA	585092	90750	0.9964	49.06–5000	15.80	47.43	3.95	11.85	2.4–8.9	0.89
sulfacetamide Na	SFC	1590160	1393	0.9989	8.7–5000	2.9	8.7	0.7	2.2	1.2–7.6	1.4
spectinomycin	SPEC	55623	−8960	0.998	173.2–5000	57.8	173.2	14.5	43.4	0.64–7.0	1.3
streptomycin	STREP	20759	−23309	0.9983	533.4–5000	177.8	533.4	44.5	133.4	0.60–6.3	1.4
sulfacarbamide	SFC	41286	−8452	0.9966	2.07–5000	0.69	2.07	0.17	0.52	2.1–3.3	0.40
sulfadiazine	SDA	5002325	−12328	0.9995	19.39–1000	6.46	19.39	1.62	4.85	0.36–5.7	0.89
sulfaguanidine	SGA	1264343	−1292	0.999	10.03–5000	3.34	10.03	0.84	2.51	0.80–12	0.87
sulfamerazine	SMA	7308819	−14598	0.9998	8.45–1000	2.82	8.45	0.71	2.12	0.84–6.1	0.66
sulfamethazine	SMZ	583452	2984	0.9863	0.08–5000	0.03	0.08	0.01	0.02	1.0–8.8	0.75
sulfamethoxazole	SMX	8078106	2984	0.9995	0.38–1000	0.13	0.38	0.03	0.10	1.7–8.2	0.89
sulfanilamide	SNA	53617	−25963	0.9973	1500.6–5000	502.9	1508.6	125.7	377.2	0.39–2.7	1.4
sulfathiazole	STA	4356784	615	0.9996	6.5–1000	2.2	6.5	0.6	1.7	0.64–6.0	1.1
tetracycline	TET	2063937	−33350	0.9990	57.2–1000	19.1	57.2	4.8	14.3	0.50–10	1.6
trimethoprim	TMP	26514921	74017	0.9994	2.9–500	0.98	2.9	0.2	0.7	1.7–3.7	1.1

**Table 3:** Antibiotic resistance of microbial strains isolated from soil collected in three different zones around the poultry farm building. A (0–50 m around the poultry farm building), B (approx. 500 m from poultry farm building), and C (approx. 1000 m from the poultry farm building). Inhibition zones around different antibiotics: trimethoprim (TMP) 5 µg, sulfametazine (SMZ) 20 µg, tetracycline (TET) 10 µg, spectinomycin (SPEC) 25 µg, and ciprofloxacin (CIP) 10 µg, are presented as average diameter (mm) of inhibition zone with standard deviation for different soil sampling zones.

Soil Samples Localization		A	B	C
N. of Strains Tested		66	15	4
Antibiotics	CIP	29.0 ± 7.5	28.8 ±5.2	28.0 ± 4.0
	SPEC	6.8 ± 8.0	6.2 ± 8.6	0.0
	SMZ	2.5 ± 7.1	0.0	0.0
	TET	13.1 ± 11.2	12.6 ± 10.1	9.5 ± 7.0
	TMP	2.7 ± 8.2	3.7 ± 9.8	0.0

## Supplementary Materials

Supplementary Materials are available online.
