# Supplementary figures and images for: Poultry Farms as a Potential Source of Environmental Pollution by Pharmaceuticals
Source: Molecules. 2020 Feb 25;25(5):1031. doi: 10.3390/molecules25051031 (PMC7179153; doi:10.3390/molecules25051031)

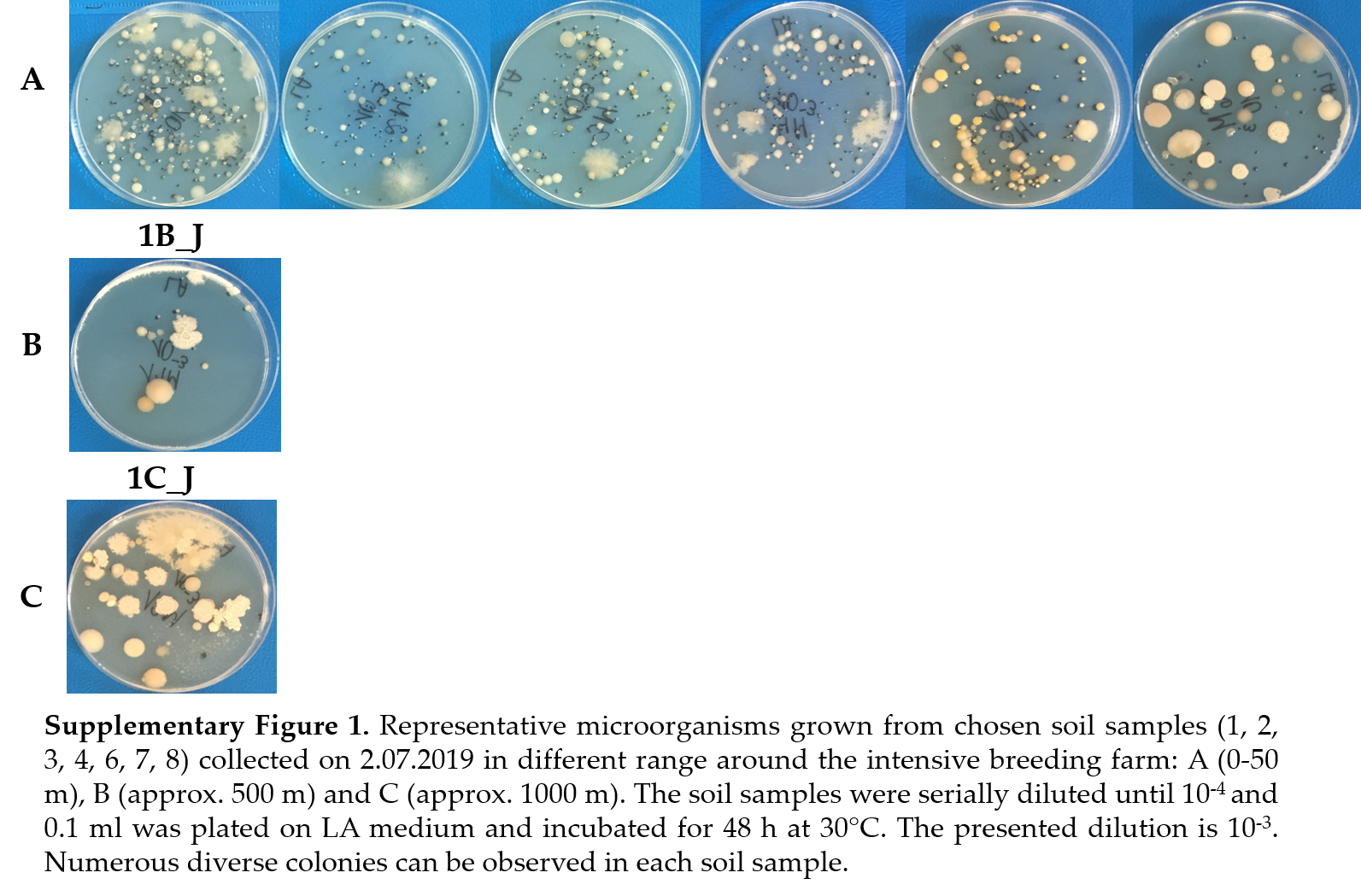

Supplement: Supplementary file 1 [file molecules-25-01031-s001.zip › molecules-727454-supplementary.tif]
